# Monoclonal antibody therapies targeting immune checkpoints induce fatal anaphylactic reactions in a murine model of breast cancer

**DOI:** 10.1186/2051-1426-2-S3-P111

**Published:** 2014-11-06

**Authors:** Christine Mall, Gail D Sckisel, Annie Mirsoian, Steven K Grossenbacher, William J Murphy

**Affiliations:** 1 Department of Dermatology, University of California, Davis Medical Center, Sacramento, CA, USA

## 

Co-inhibitory molecules such as PD1, PD-L1 and CTLA-4 are being increasingly used as targets of therapeutic intervention against cancer. The use of monoclonal antibodies targeting these immune checkpoints has been shown to promote anti-tumor immune responses clinically. While these promising results have led to a critical paradigm shift in treatments for cancer, these approaches are also plagued with limitations owing to cancer immune evasion mechanisms and adverse toxicities associated with continuous treatment. It has been difficult to reproduce and develop interventions to these findings preclinically for many reasons including species/age-related differences in expression of these markers, poor tumor modeling, and reagent xenogenicity. In this study, we investigated adverse effects in mice receiving repeated anti-PD1 (clone J43) or PD-L1 (clone 10F.9G2) monoclonal antibody (mAb) administration in the 4T1 mouse model of mammary carcinoma. Mice bearing day 14 syngeneic mammary carcinomas were treated with mAbs to PD1 or PD-L1 which are of rat or hamster origin respectively. Control mice received rat IgG or hamster IgG mAb. Repeat administration of 250 mg i.p. of anti-PD1 or 200 mg i.p. of anti-PD-L1 into tumor bearing mice led to mortality in 50-100% of treated mice within 10 days of the initiation of treatment whereas mice receiving the control mAbs exhibited complete survival at equivalent doses. These toxicities were observed within 1-5 hours of administration of the final dose. Consistent with anaphylaxis, symptoms including piloerection, periorbital puffiness and dyspnea were observed prior to mortality. Furthermore, spleens of mice treated with anti-PD1 were observed to be greater in size relative to untreated mice indicating increased myelopoiesis. Previously, 4T1 has been associated with high expression of granulocyte-colony stimulating factor (G-CSF) systemically resulting in increased extramedullary hematopoiesis and the accumulation of myeloid derived suppressor cells (MDSCs). Both G-CSF and MDSCs have been shown to play an exacerbating role in anaphylactic reactions. Our observations here suggest that the foreign nature of anti-PD1 and anti-PD-L1 along with their important role in checkpoint blockade combined with tumor-induced immune modulation may promote an anaphylaxis-like response in tumor-bearing mice. This study highlights the importance of species-specific mAbs for preclinical models as well as the interesting roles of both checkpoint blockade and tumor-dependent immunomodulation contributing to this particular class of cancer immunotherapy.

